# Socioeconomic inequalities and body mass index in Västerbotten County, Sweden: a longitudinal study of life course influences over two decades

**DOI:** 10.1186/1475-9276-13-35

**Published:** 2014-05-07

**Authors:** Mojgan Padyab, Margareta Norberg

**Affiliations:** 1 Centre for Population Studies, Ageing and Living Conditions Programme, Umeå University, Umeå, Sweden; 2 Epidemiology and Global Health, Department of Public Health and Clinical Medicine, Umeå University, Umeå, Sweden

**Keywords:** Socioeconomic inequality, Life course, Body mass index, Longitudinal study, Northern Sweden

## Abstract

**Introduction:**

Life course socioeconomic inequalities in heart disease, stroke and all-cause mortality are well studied in Sweden. However, few studies have sought to explain the mechanism for such associations mainly due to lack of longitudinal data with multiple measures of socioeconomic status (SES) across the life course. Given the population health concern about how socioeconomic inequality is related to poorer health, we aim to tackle obesity as one of the prime suspects that could explain the association between SES inequality and cardiovascular disease and consequently premature death. The aim of this study is to test which life course model best describes the association between socioeconomic disadvantage and obesity among 60 year old inhabitants of Västerbotten County in Northern Sweden.

**Methods:**

A birth cohort consisting of 3340 individuals born between 1930 and 1932 was studied. Body mass index (BMI) at the age of 60 and information on socioeconomic status at three stages of life (ages 40, 50, and 60 years) was collected. Independent samples *t*-test was used to compare BMI between advantaged and disadvantaged groups and one-way ANOVA was used to compare BMI among eight SES trajectories. We applied a structured modeling approach to examine three different hypothesized life course SES models (accumulation, critical period, and social mobility) in relation to BMI.

**Results:**

We found sex differences in the way that late adulthood socioeconomic disadvantage is associated with BMI among inhabitants of Northern Sweden. Our study suggests that social adversity in all stages of late adulthood is a particularly important indicator for addressing the social gradients in BMI among women in Northern Sweden and that unhealthy behaviors in terms of smoking and physical inactivity are insufficient to explain the relationships between social and lifestyle inequalities and BMI.

**Conclusion:**

In order for local authorities to develop informed preventive efforts, we suggest further research to identify modifiable risk factors across the life course which could explain this health inequality.

## Introduction

It is well-established that obesity is a major risk factor for mortality and morbidity and is on the rise globally [1]. This topic calls for more attention from public health researchers and politicians in Sweden since evidence has shown that obesity is increasingly prevalent in Sweden overall [2], as well as Northern Sweden, where the absolute prevalence of obesity increased from 4.9% in 1990–1995 to 10.3% in 2002–2007 among men and from 3.2% to 6.5% among women [3]. In a population-based study among Swedish women aged 30–50 years, Power and his colleagues (2005) examined whether childhood and adulthood socioeconomic status (SES) influenced adult obesity [4]. The results showed that three times as many women in manual occupations in adulthood were obese, compared to those in non-manual occupations, after adjusting for childhood SES. Results from a previous study among Swedish women 45–73 years old indicated that women with low educational attainment or low SES (both in their own adulthood or their parents’ SES at childhood) had higher weight gain (weight gain between young adulthood at the age of 20 and current weight) compared with women from higher education or higher SES background [5].

While the evidence for the contribution of life course SES to the development of weight change or obesity is generally strong [6], it remains unclear which period of late adulthood is more concerning. The time is ripe to go beyond the simple dichotomy of childhood versus adulthood and take the opportunity to have multiple time point exposures.

There has been a burgeoning literature in recent decades on the association between socioeconomic factors and total mortality and CVD risk factors in life course analysis using multiple measurements [7,8]. This can be achieved by repeated measures of socioeconomic position from early childhood into adult life [9]. Because CVD is a major cause of morbidity and mortality among older people [10] it is sensible that research seek out possible mechanisms that may explain the association between life course socioeconomic inequalities and cardiovascular diseases. Body weight is the prime suspect and it is potentially an ideal modifiable risk factor as body weight tends to increase during the life span throughout late middle age [11,12].

Socioeconomic differences in mortality are well documented in most European countries including Sweden [13]. People from a lower economic class are at higher risk of cardiovascular disease (CVD) risk factors, morbidity and early death [14-16]. Both in Sweden and other countries, there is evidence on geographical inequalities in mortality and morbidity due to differences in social and material deprivation and the association between poor living conditions and health may differ by geographical context and socioeconomic conditions [17,18]. Owing to extensive epidemiological studies on cardiovascular disease (CVD) in Northern Sweden in 1980, it became apparent that Västerbotten county had developed the highest CVD mortality in Sweden [19]. Even though from 1985–1999 this population had an annual decrease in incidences of fatal and nonfatal acute myocardial infarction reported [20], findings from public health research in this specific region would benefit the local community and facilitate local policy makers with informed decisions.

Various life course hypotheses on the influence of life course SES on health outcomes have been proposed, which can be grouped into three broad conceptual models: the *accumulation of risk model*, the *critical period model* and the *social mobility model*. The most prominent model in the current literature is the *accumulation of risk model*, which assumes that cumulative adverse exposures during the life course increases the risk of CVD risk factors irrespective of the timing [9,21]. The accumulation model suggests that exposures across the life course accumulate (through for example periods of adverse social and environmental conditions) and may have adverse effects on health in the longer term. Alternatively, the *critical or sensitive period life course model* focuses on the timing of an exposure and assumes that adverse exposures in a specific time period have a negative effect on health outcomes, with no (or less) disease risk outside of that specific period [22]. The *social mobility* model has been less strictly defined than the accumulation and critical period models but it generally implies that the irreversible effect of the critical period can be either enhanced or diminished by later effects [23]. Pollitt and colleagues [22] have suggested that *social mobility* across the life course has an impact on CVD mortality risk factors. The results, which are based on the comparison of stable high, stable low, upward mobility, and downward mobile groups, provide evidence that the CVD risk of the socially mobile groups are intermediate compared to the two stable groups.

The current study will shed some light on this topic in Northern Sweden and examine three life course models, corresponding to the accumulation, critical period, and social mobility in relation to body mass index in later life. Specifically, the primary aim will be to examine whether social disadvantage at each phase, and cumulatively across, the late adulthood has an impact on body mass index after a 20 year follow-up. We will investigate these associations separately for men and women. In addition, we aim to investigate if the SES-body mass index association could be explained by health behaviors.

## Methods

### Setting

Västerbotten County is located in Northern Sweden and occupies one eighth of the country, from the Gulf of Bothnia to the Scandinavian Mountains. The population of approximately 260,000 is concentrated on the coast and about 45% live in the city of Umeå. Umeå is a medium-sized city and is home to the regional administrative center, a university, and the regional hospital. A third of the population lives in two small cities (Skellefteå and Lycksele) and the rest of the population is spread out into smaller municipalities.

### Data: the Linnaeus database

Data originated from the Linnaeus database, available at the Centre for Population Studies, Umeå University, Sweden [24]. The Linnaeus database is constructed by linking individual records from multiple national sources with two local datasets: Västerbotten Intervention Programme (VIP) and Betula [24]. The national registers include the Death Cause Register (death and cause of death), Inpatient Register (hospitalization and diagnosis), and Statistics Sweden. A large part of the data from Statistics Sweden comes from the Longitudinal integration database for health insurance and labor market studies (LISA by Swedish acronym) and also from Censuses, the Multigenerational Register and the Geography Database. Civic ID numbers are replaced by codes, and the key is preserved by Statistics Sweden.

### Västerbotten intervention programme (VIP)

The VIP has been conducted in Västerbotten County in Sweden since 1985 as a response to high cardiovascular disease (CVD) mortality rate in the county, which was the highest in the country. It was planned in collaboration with health care providers and Umeå University, piloted in a small community and then launched in the whole county in 1990. All inhabitants are invited to a health examination during the years in which they turn 40, 50, and 60 years old. The results of the examinations are discussed individually with each participant following the principles of motivational interviewing. More details about VIP have been reported elsewhere [25].

### Subjects

A cohort of all inhabitants aged 60 years, who participated in the Västerbotten Intervention Program [25] during 1990–1992, were chosen as the study population. A retrospective cohort study design was used where BMI records were taken from VIP during 1990–1992 and the study involved looking back at registered SES available from Statistics Sweden. Participants were 38–40 years old in 1970, 48–50 years old in 1980 and 60 years old in 1990–1992.

### Measures

#### Outcome: body mass index (BMI)

Height and weight were measured in light indoor clothing and used to calculate BMI (kg/m^2^). BMI is considered as a continuous and normally distributed variable. A BMI greater than or equal to 25 is overweight and greater than or equal to 30 is obesity.

#### Exposure: SES over the late adulthood

The Socioeconomic Status was classified by Statistics Sweden and was measured at three time points at the ages of 40, 50 and 60 years old. We applied the seven categories of SES by Statistics Sweden: (I) Self-employed professionals (II) Non-manual employees at higher level (III) Non-manual employees at intermediate level (IV) Non-manual employees at lower level (V) Self-employed other than professionals and farmers (VI) Skilled workers (VII) non-skilled workers. For the purpose of this study, binary indicators of SES were created at each time point by collapsing the SES measures into two categories: advantaged (categories I-V) was given a value of 0, and disadvantaged (categories VI-VII) was given a value of 1.

Farmers are excluded in this study.

#### Potential mediators: health behaviors

Information on health behaviors at the age of 60 was obtained by routine questionnaires available from the VIP. It included current daily smoking (yes = 1/no = 0) and physical activity (inactive = 1, active = 0) [26].

### Statistical analysis

BMI at the age of 60 years old is the outcome and is treated as a continuous variable. Independent samples *t*-test was used to compare BMI between advantaged and disadvantaged groups and one-way ANOVA was used to compare BMI among eight SES trajectories. We applied a structured modeling approach developed by Mishra et al. [23] to examine three different hypothesized life course SES models (accumulation, critical period, and social mobility) in relation to BMI. Based on this approach, a partial F-test will be used to compare the saturated univariate ANOVA model (all main effects and interactions) with reduced models.

First, a saturated model was calculated and the model assumes that each of the possible trajectories is associated with a different value of the outcome. Second, partial F-tests were applied to compare the saturated model with reduced models, nested within the saturated model, indicating a selected effect corresponding to each life course model. A significant F-test indicates that the reduced model shows a poor fit compared to the saturated model and therefore did not support the specific life course model, whereas large p-values indicate that the more parsimonious, nested model provided an adequate support for the specific life course model.

Because of potential differences in association of SES with BMI between men and women, all analyses are stratified by sex. All analyses are performed using STATA version 12.0 (StataCorp, College Station, TX).

### Ethical considerations

This study was approved by the regional Research Ethics Board in Umeå (08-131M and 07-142Ö). Individuals gave informed consent prior to each health screening.

## Results

The data consisted of 1522 (46%) men and 1818 (54%) women. The characteristics of the study population and distribution of socioeconomic trajectories among men and women of VIP participants are given in Table 1.

**Table 1:** Characteristics of the study population and distribution of socioeconomic trajectories among VIP participants for the years 1990–1992, by sex

**Characteristics**			**Men n = 1522**	**Women n = 1818**
**Smoking**^**a**^				
Current daily smoking (%)			23.0	19.3
**Physical activity**^**a**^				
Active (%)			38.6	40.6
**SES at the age of 40 (%)**				
Advantaged (0)			34.2	34.0
Disadvantaged (1)			65.8	66.0
**SES at the age of 50 (%)**				
Advantaged (0)			51.9	45.3
Disadvantaged (1)			48.1	54.7
**SES at the age of 60 (%)**				
Advantaged (0)			56.3	44.1
Disadvantaged (1)			43.7	55.9
**SES40**	**SES50**	**SES60**		
0	0	0	34.6	28.1
1	0	0	15.9	14.6
0	1	0	1.3	1.3
0	0	1	1.7	2.0
1	1	0	5.0	2.7
1	0	1	3.3	2.9
0	1	1	2.9	9.3
1	1	1	35.3	39.1

^a^health behaviors concurrent with the BMI at the age of 60.

About 35% of the men population had stayed in the advantaged group at all three time points (stable advantaged) which was the same for the stable disadvantaged group. Among women, there were about 40% who stayed in the stable disadvantaged group whereas a lower proportion (28%) stayed in the stable advantaged group (Table 1).

Trajectories that indicated change in SES at three stages of life (ages 40, 50 and 60 years) were dominated by those who moved from disadvantaged at 40 to the advantaged group by the age of 50 and remained there until they were 60 years of age (15.9% for men and 14.6% for women). Those women who were always in the disadvantaged group had a significantly higher BMI compared to those who were continuously in the advantaged group (26.6 vs. 25.3 kg/m^2^, P < 0.01, Table 2).

**Table 2:** Mean BMI ± standard deviation of the study population by socioeconomic status (SES) in men (n = 1522) and women (n = 1818)

**Method of describing SES**			**Men**	**Women**
*Time period individually*				
**SES at the age of 40 (t**_**1**_**)**				
Advantaged (0)			26.4 ± 3.3	25.7 ± 3.8^*^
Disadvantaged (1)			26.4 ± 3.4	26.7 ± 4.4
**SES at the age of 50 (t**_**2**_**)**				
Advantaged (0)			26.4 ± 3.3	25.7 ± 3.8^*^
Disadvantaged (1)			26.3 ± 3.5	26.8 ± 4.4
**SES at the age of 60 (t**_**3**_**)**				
Advantaged (0)			26.4 ± 3,3	25.6 ± 3.5^*^
Disadvantaged (1)			26.2 ± 3.5	26.6 ± 4.4
*SES trajectories across three time periods*				
t_1_	t_2_	t_3_		
0	0	0	26.4 ± 3.5	25.3 ± 3.6^**^
1	0	0	26.4 ± 3.1	25.8 ± 3.4
0	1	0	26.1 ± 2.8	25.3 ± 3.1
0	0	1	26.5 ± 3.6	25.7 ± 4.9
1	1	0	25.9 ± 3.1	26.6 ± 3.1
1	0	1	26.3 ± 3.2	26.3 ± 3.4
0	1	1	25.5 ± 2.8	26.1 ± 3.8
1	1	1	26.1 ± 3.5	26.6 ± 4.4
*Accumulation score: number of times ‘disadvantaged’*				
0			26.4 ± 3.5	25.3 ± 3.6 ^†^
1			26.4 ± 3.1	25.8 ± 3.5
2			25.9 ± 3.1	26.2 ± 3.6
3			26.1 ± 3.5	26.6 ± 4.4

*P < 0.01 compared to the disadvantaged group.

**P < 0.01 compared to the stable disadvantaged group (111).

^†^P < 0.01 compared to the most disadvantaged group (n _disadvantaged_ = 3).

Alternative univariate ANOVA models were fitted to the data corresponding to the hypotheses for the effect of SES over the life course. A full factorial univariate ANOVA was performed for BMI including the three SES indicators and their interactions. Next, partial F-tests corresponding to different life course hypotheses were applied to compare the full factorial model with reduced models. We also compared the saturated model with the null model (i.e., a model including only the intercept).

In men, the null model produced a non-significant p-value when compared with the saturated model, suggesting that SES was not associated with BMI among men and therefore we did not proceed with further analysis. Among women, partial F-tests were non-significant (all p-values >0.1) for accumulation of risk hypothesis and critical period, suggesting that both life course models explained the data equally well as the saturated model (Table 3). For women, the accumulation of risk model indicated a significant increased BMI of 0.44 kg/m^2^ (95% confidence interval: 0.26 to 0.62) for a unit increase in SES score (number of times 0–3 in disadvantage group). Critical period models at the ages of 40, 50 and 60 showed non-significant p-values (Table 3) with an effect of disadvantage group on BMI of 1.05 (95% CI: 0.63 to 1.47), 1.12 (95% CI: 0.68 to 1.56), and 0.96 (95% CI: 0.52 to 1.39) kg/m^2^ for critical times t_1_, t_2_, and t_3,_ respectively.

**Table 3:** Partial F tests for different contrasts according to different hypotheses among women

	**Model 0: Bivariate**			**Model 1:+ health behaviors**		
**Life course model**	***df***_***1***_**,** *** df***_***2***_	***F- statistic***	***P-value***	***df***_***1***_**,** *** df***_***2***_	***F- statistic***	***P-value***
*Accumulation of risk*	6, 1110	0.11	0.9	6, 1061	0.06	0.9
*Critical period*						
t_1_ (SES at the age of 40)	6, 1110	1.37	0.2	6, 1061	1.67	0.1
t_2_ (SES at the age of 50)	6, 1110	1.08	0.4	6, 1061	1.16	0.3
t_3_ (SES at the age of 60)	6, 1110	1.14	0.3	6, 1061	1.34	0.2
*Social mobility*						
Late life mobility	5, 1110	4,59	<0.001	5, 1061	5.10	0.001
Any mobility	5, 1110	4.56	<0.001	5, 1061	5.09	0.001

Predictors in Model 0 = SES corresponding to each life course model.

Predictors in Model 1 = Model 0+ health behaviors concurrent with the outcome (Physical activity and smoking).

The social mobility models (‘Late life mobility’ and ‘Any mobility’) showed particularly poor fits as they were significantly different from the saturated model (P < 0.001, Table 3). The late social mobility (t_2_ to t_3_) model estimated no change in BMI for upward (U) adult SES mobility [U_23_ (95%CI): −0.26 (−1.3 to 0.82), P = 0.8], as well as downward (D) adult SES mobility [D_23_ (95%CI): 0.01 (−1.21 to 1.32), P = 0.9].

## Discussion

This study extends the literature on the association between SES inequality at different stages of one’s adulthood and body mass index in the county of Västerbotten, Sweden. Using the structured approach, we considered the whole spectrum of alternative model specifications, i.e., accumulation model, critical period model, and social mobility model. A previous study on life course SES and body mass index was conducted in Northern Sweden, in which SES was obtained at the ages of 16, 21, 30, and 43 years [27]. The major contribution of our study is in the extension of life course multiple measures of SES from the ages of 40 to 60 years, which eventually can give a complete picture of this process in Northern Sweden. We demonstrated that in women but not in men, associations between SES and BMI across the life course correspond to both accumulation and critical period models and that behavioral factors, including smoking and physical activity, do not mediate this association. It has been found that the likelihood of finding an association between SES and BMI in men increased when prospectively collected SES was used [7], suggesting null SES impact on BMI may be a result of recall bias. Our results show that the absence of life course SES association with BMI is less likely due to recall bias because the risk of recall bias is minimized when information on SES was collected from a registered database (Statistics Sweden). Results from the prospective Northern Swedish Cohort study [27] corroborate our findings, revealing non-significant associations in men, although health behaviors contributed strongly to body mass index.

We have used a new structured approach to investigate theoretical life course models of SES that are frequently applied in the literature [7,23,28]. This approach can be used within a regression framework as well as generalized linear models (e.g., Poisson or logistic) with the only difference concerning the test statistics used to compare nested models with a more general (saturated) model. While a partial F-test should be used in linear regression to compare nested models, the likelihood ratio test or one of its approximations would be used with generalized linear models [29].

The current study examines the association between life course SES and BMI with specific focus on late adulthood SES trajectories. By using the structured approach, we found sex differences in the way late adulthood life course SES is associated with body mass index among inhabitants of Northern Sweden in their 60s. The present findings confirm that in women, the association between late adulthood SES at the ages of 40, 50 and 60 and body mass index is consistent with two life course models, namely the accumulation and critical period models. While most previous research has included childhood and adolescent SES and adult BMI, the current study focuses on late adulthood social differentials and its association with body mass index later in life. In concordance with previous literature, we did not find evidence for life course associations between SES and BMI among men [6,7,27].

A review of the literature on the association between childhood socioeconomic position and adult obesity [6] revealed that such association is less consistent among men, with only 3 of 14 studies [30-32] showing that childhood SES has an effect on adult body mass index, after adjusting for age and adult SES. While such studies focus on childhood SES more, we found no evidence of such associations in late adulthood among men. This suggests that the situation is not all that different in earlier stages of adult life SES and body mass index later in life. This means that the larger amount of weight gain occurs at earlier stages of life, at least before late adulthood, and that SES has no further impact on body mass index after early adulthood among men.

There are potential mechanisms that may explain the sex differences in the association between life course SES and adulthood obesity. Such mechanisms include parity, socioeconomic-patterned pressure to be slim among women and the link between occupational choice and wage with obesity among women [6,33]. With regard to parity, there is convincing evidence that women’s educational attainment is inversely related to birth rate [34,35]. Even more specifically in developed countries of Northern Europe and postindustrial countries including Sweden, completed fertility of the cohorts of women born in the 1930s (to whom, the cohort of our study belong) and the 1940s is negatively associated with educational level [36]. As childbirth is associated with increased long-term central obesity [37], this could explain why SES-obesity association is stronger among women in our cohort than men. However, this argument needs to be tested for Swedish cohorts of women born after 1930 as there is evidence that the association between education and fertility fades substantially in later cohorts [38].

There is evidence to suggest that there may be stronger social pressure against obesity in women compared with men, and the pressure is stronger among women at higher socioeconomic status than among women at low socioeconomic status [33,39]. Although enough research on attitudes toward body weight has been conducted on young women, available reports suggest that body image dissatisfaction and weight preoccupation remain high in aging women [40]. It has been shown that sex differences regarding eating habits, body weight, and physical appearance are evident across the life span, with women of all ages displaying more weight-related concern than men [40]. Some evidence suggests that obesity, primarily among women, is associated with individuals’ occupation. Several reports from the National Longitudinal Survey of Youth (NLSY) found no statistical significant effect of wages on men’s weight but that higher wages are associated with lower weight among women [41,42] . However, the direction of the association between wages and weight remains unclear in those studies.

The social mobility models in women showed particularly poor fits as they were significantly different from the saturated model (p < 0.001). The general social mobility model assumes that all downward changes are equally harmful to health and that all upward changes are equally beneficial [23]. This means there is the same expected BMI in those with an SES which never changed, and in those who moved from non-manual to manual occupations or in those who moved from manual to non-manual occupations at some point between 40 to 60 years old. This hypothesis was rejected in our study as there was a significant difference between the social mobility model compared to the saturated model and also there was an evident increased BMI among women who remained in non-manual occupations at three stages of life compared to women who remained in manual occupations at all three points. Small numbers of socially mobile individuals could also explain the non-significant results of the mobility models.

We note that smoking and physical activity are only two lifestyle aspects that are included in the analysis. Unfortunately, data on energy intake and food habits was unavailable. Even though we used a validated instrument to measure physical activity, it would be more accurate if it were not self-reported. Unhealthy behaviors including lack of physical activity [43] and smoking [44] tend to be higher in adults with low SES compared with those with a high SES in the county of Västerbotten. While this inverse gradient would contribute the social gradient in obesity, our results did not provide any evidence on the mediating role of such behaviors on the association between life time SES and obesity. This finding is consistent with findings from the Northern Swedish Cohort [27] study and suggests that non-behavioral mediators might play a stronger role. For example, socially-disadvantaged women, to a greater extent than men, are susceptible to disturbances in the physiological stress system after experiencing an unfavorable situation over the life course and that might lead to adiposity later in life. It is noteworthy that cohort effect might be present in the present study. The early VIP data show that today, BMI among 40 year olds is similar to those of 60 year olds while in the 1990s the 40-year old had considerably lower BMI compared to those who were 60 years old in 1990.

## Conclusion

Our study suggests that social adversity at all stages of late adulthood is a particularly important indicator for addressing the social gradients in body mass index among women in Northern Sweden and that unhealthy behaviors in terms of smoking and physical inactivity are insufficient to explain such association. In order for local authorities to develop informed preventive efforts, we suggest further research to identify modifiable risk factors across the late adulthood which could explain this health inequality. Our results suggest that efforts should be concentrated on reducing the social inequality that is associated with obesity in women in mid-adulthood and that such efforts should not be limited to targeting unhealthy behaviors.

## Competing interests

The authors declare that they have no competing interests.

## Authors’ contributions

MN and MP participated in the design of the study. MP performed the statistical analysis and drafted the manuscript. Both authors read and approved the final manuscript.
